# Anti-dyskinetic efficacy of 5-HT3 receptor antagonist in the hemi-parkinsonian rat model

**DOI:** 10.1016/j.ibror.2018.12.001

**Published:** 2018-12-14

**Authors:** Nazanin Aboulghasemi, Mahsa Hadipour Jahromy, Amir Ghasemi

**Affiliations:** a Herbal Pharmacology Research Center, Faculty of Medicine, Tehran Medical Sciences, Islamic Azad University, Tehran, Iran; b Dept. of pharmacology, Faculty of Medicine, Islamic Azad University, Tehran Medical Sciences, Tehran, Iran; c Department of Pharmacology, Faculty of Medicine, Tehran Medical Sciences, Islamic Azad university, Tehran, Iran

**Keywords:** Dyskinesia, Levodopa, Parkinson, Ondansetron, Serotonin, Rat

## Abstract

Parkinson's disease is a progressive debilitative neurodegenerative disease characterised mostly with bradykinesia, tremor, catatonia, drooping posture, unsteady gate and unstable steps. Levodopa has been proven to be among the most effective and acceptable treatment that can reconstitute dopamine in Parkinson's disease. However, there is a relation between levodopa long term administration and dyskinesia. Regarding the effectiveness of ondansetron in Parkinson's disease, we planned to test its effect on levodopa-induced dyskinesia (LID). In this study, Parkinsonism was induced in 40 adult male rats using 6-OHDA injection into the striatum via stereotaxic surgery. After 2 weeks, all animals tested for Parkinson's disease using apomorphine rotation test. Then, animals with positive symptoms for Parkinsonism divided into 4 equal groups, the first group treated with levodopa 50 mg/kg i.p, the second group received only distilled water, the third and forth groups treated with levodopa 50 mg/kg i.p plus two different doses of ondansetron (0.04 and 0.08 mg/kg i.p) for 3 weeks. Animals tested for dyskinesia using AIMs and rotarod tests at specific days and a week after discontinuation of ondansetron. Evaluations of AIMs test showed significant changes in dyskinetic movements and reduction in scores in groups treating with ondansetron when compared with the first group. Upon discontinuations of ondansetron in the last two groups, AIMs scores significantly increased. While in rotarod test, ondansetron had no additional benefit when added to levodopa in motor coordination of animals. Findings of this study suggest that co administration of ondansetron with levodopa is effective in attenuating dyskinesia.

## 1 Introduction

During long term treatment of Parkinson's disease (PD) with Levodopa, serious dyskinetic movements have been reported that is known as Levodopa-induced dyskinesia (LID). The management of LID is a challenging issue. It has serious negative effects on the quality of the patient’s life and limits the use of levodopa that is the most effective drug for control of the disease (Guridi et al., 2012).

In the last few decades, research interest has been focused on medications that could provide more continuous dopaminergic stimulation (Yang et al., 2012). However, this strategy was not successful and the control of LID remained an unsolved problem. Therefore, recent findings regarding the pathophysiology of LIDs changed towards non-dopaminergic pathways as possible targets for modulating the emergence of LIDs. Among different pathways, it seems that serotonergic neurotransmission plays an important role in dopaminergic function (Yang et al., 2012; Stansley and Yamamoto, 2015; Marty et al., 1990; Ohno et al., 2013a, 2013b). It is proposed that inhibition of some subtype receptors of serotonin like 5-HT2 A/2C, 5-HT3 and 5-HT6 can improve extrapyramidal disorders (Ohno et al., 2015).

Specifically, it is also reported that stimulation of 5-HT1A receptors is effective for multiple PD symptoms including Parkinsonism, levodopa-induced dyskinesia (LID), cognitive impairment, mood disorders and neuro-degeneration of dopamine neurons (Ohno et al., 2015; Carta and Tronci, 2014). Blockade of 5-HT2 receptors is also likely to improve Parkinsonism, depressive mood and cognitive impairment. In addition, it was recently demonstrated that 5-HT2 A inverse agonists can alleviate PD psychosis. The involvement of the serotonin neurons in the appearance of LID has also been demonstrated in a rat PET-imaging study (Zoldan et al., 1995; Sirota et al., 2000; Stockmeier et al., 1993).

Ondansetron is a highly specific and selective serotonin 5-HT3 receptor antagonist, marketed under the brand name Zofran, is a medication used to prevent nausea and vomiting caused by chemotherapy of cancer, surgery and morning sickness. It has low affinity for dopamine receptors (Deegan, 1992). The 5-HT3 receptors are present both peripherally on vagal nerve terminals and centrally in the chemoreceptor trigger zone of the area postrema (Hannon & Hoyer, 2008; Houtani et al., 2005). It is found that ondansetron can be useful in ameliorating antipsychotic-induced tardive dyskinesia in people with schizophrenia, and the reports showed significant improvement in the disease's symptoms (Zoldan et al., 1995; Sirota et al., 2000).

No report has been published about possible effectiveness of ondansetron as a 5-HT3 antagonist on LID, so far. We proposed that serotonergic neurotransmission might affect dopamine release in striatum possibly via 5-HT3 receptor stimulation. To test this hypothesis, the first in vivo study of potential effects of 5-HT3 antagonist in levodopa-induced dyskinesia was planned and we tried to evaluate its role on LID in 6-Hydroxydopamine (6-OHDA) model of Parkinson's disease in rat.

## 2 Materials and methods

### 2.1 Animals

Forty male Wistar rats (Pasteur Institute, Tehran, Iran), weighing 175–200 g were used to induce Parkinsonism and divided in four equal groups. A separate group of ten animals were selected to receive vehicle in striatal injection as control. All animals maintained in temperature-controlled conditions with a 12 h light/ dark cycle (lights off from 6:00 A.M. to 6:00 P.M.). Water and food were provided *ad libitum* except for testing periods. The ethical and legal approval for animal experiments of the present study has been obtained from Research and Technology Deputy of Azad University, Tehran Medical Sciences Branch, Tehran, Iran that follow in accordance with the National Institutes of Health guide for the care and use of Laboratory animals (NIH Publications No. 8023, revised 1978).

### 2.2 6-OHDA-lesions and behavioral screening

Dopamine-denervating lesions were performed by unilateral injection of 6- OHDA into the striatum. All Rats were anaesthetized with Ketamine hydrochloride and Xylazin (50 mg/kg and 5 mg/kg, i.p., respectively) and mounted on a stereotaxic frame (Kopf Instruments, Tujunga, CA, USA). Forty animals received 6-OHDA-HCl (Sigma-Aldrich Sweden AB) that was dissolved in 0.02% ascorbate ± saline (vehicle) at a concentration of 3 mg/ mL free base 6-OHDA. Vehicle injection was performed for one separate group. Injections were done at the rate of 0.5 micro L/min (the needle were kept for additional 2 min at site before retracting) using a 10-MicroL Hamilton microsyringe with a 26-gauge steel cannula (Supa. Co, Iran). Stereotaxic coordinates into the striatum were 1.0 mm anterior to the bregma, 3.0 mm left of the midline, and 5.5 mm ventral to the dura, and the tooth bar was set at -3.3. In order to assess the efficacy of the lesions, all rats were tested for apomorphine-induced rotation at 2 weeks after the 6-OHDA injections (Cui et al., 2014). After an i.p. injection of apomorphine hydrochloride (0.5 mg/kg, dissolved in saline), net full body turns per min were considered, where rotation towards the side of the lesion was given a positive value. Rats that showed rotational scores > 4 net full turns/min in the direction ipsilateral to the lesion were kept in the study. Total net ipsilateral rotations were measured in 5 min. In our experiments, only two rats couldn't pass the test and removed from the study. Also, one rat died after cannula insertion.

### 2.3 Abnormal involuntary movements (AIMs) test

In test days, animals were placed in a cage (30 × 20 × 20 cm) on a mirror to observe any abnormal involuntary movements in a total time of 3 h with 30 min intervals for 1 min each time (totally, seven times in 3 h). Abnormal involuntary movements included axial, orolingual, limbs and locomotor movements were considered. Severities of 0–4 were assigned for each movement. A score of 0 was assigned for the absence of AIMs; 1 for occasional AIMs (less than 50% of observation time); 2 for frequent AIMs (more than 50% of observation time); 3 for AIMs that were continuous but interrupted by strong sensory stimuli; and 4 for continuous, uninterrupted AIMs. For all AIMs category, the scores were summed in each time point and then the average of multiple observations are calculated and reported.

### 2.4 Rotarod test

This is a test of motor coordination that compares the latency to fall on the multiple trials between groups. The latency to fall from a rotating rod is scored automatically with infrared sensors in a Rotamex 5 rotarod (Columbus Inst; Columbus, Ohio).

This test was performed on 3 consecutive days and in 2 sessions per day. Each session lasted 120 s, during which the rotating rod underwent a linear acceleration from 3 to 30 rpm. The amount of time that the animals were capable of stepping on the rotating rod was a criterion of their performance ability.

### 2.5 Experimental design

**Fig. 1:**
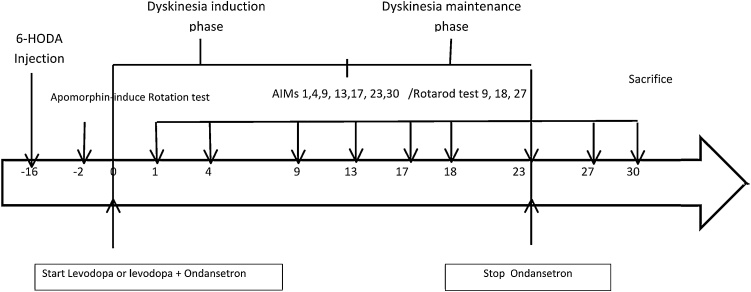
Design of the experimental study.

First, Parkinsonism was induced in 40 adult male rats using 6-OHDA injection into the striatum via stereotaxic surgery. After 2 weeks, all animals tested for Parkinson's disease using apomorphine rotation test. Then, animals with positive symptoms for Parkinsonism were divided into 4 equal groups, the first group received levodopa in distilled water 50 mg/kg i.p (Ramopharmin Pharmaceutical Co, Iran), the second group received only distilled water, the third and fourth groups were treated with levodopa 50 mg/kg i.p plus two different doses of ondansetron (Exir Pharmaceutical Co., Iran) in distilled water 30 min before levodopa treatment (0.04 and 0.08 mg/kg i.p) for 23 days (the lower dose, 0.04 mg/kg was chosen based on the report of its usefulness in controlling Tardive Dyskinesia in human: 12 mg/day) (Sirota et al., 2000). Animals tested for dyskinesia using AIMs tests in days 1, 4, 9, 13, 17 and 23. Rotarod test was started at day 9 (9 days after starting levodopa treatment) when dyskinesia confirmed by AIMs test and again measured at nearly mid-study in day 18 and finally in day 27 that was near the end of the study (Fig. 1).

### 2.6 Statistical analysis

To analyze the results, SPSS software (24th edition) was used. In the chronic drug treatment study, comparisons of AIM and rotarod scores were performed using repeated-measures ANOVA, and post hoc comparisons were performed where appropriate using the Tukey's test.

## 3 Results

### 3.1 Apomorphin rotation test

The results of apomorphine-induced rotational test following vehicle and 6-OHDA injections in rats are reported in Table 1. The number of net rotations was significantly higher in the 6-OHDA-treated groups compared to the vehicle group. Then groups 1–4 were randomly assigned as control, levodopa, levodopa + ondansetrone 0.04 mg/kg and levodopa + ondansetrone 0.08 mg/kg.

**Table 1:** Apomorphine-induced rotational test results. Data are the mean ± S.E.M. * p < 0.001 vs. vehicle.

Groups	Net turns/5 minutes
Vehicle	3.57 ± 0.70
1; levodopa	25.15 ± 3.50*
2; Control	27.42 ± 3.75*
3; levodopa + ondansetrone 0.04 mg/kg	28.63 ± 3.05*
4; levodopa + ondansetrone 0.08 mg/kg	26.78 ± 4.56*

### 3.2 AIMs behavioral test results

**Fig. 2:**
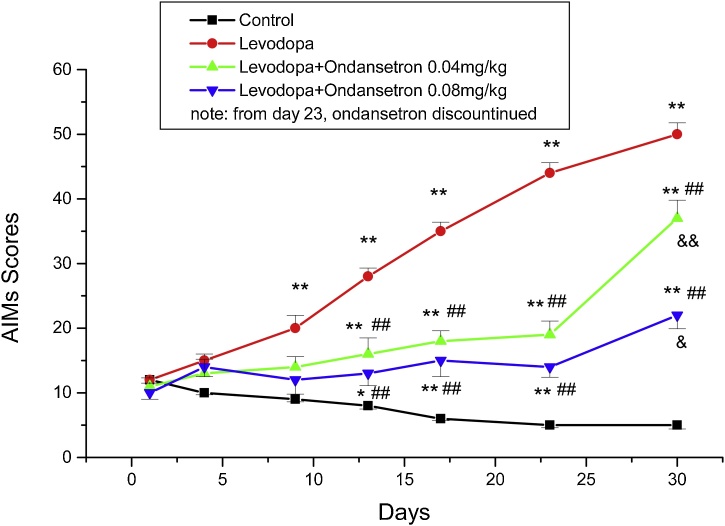
Mean AIMs scores ± SEM for four groups (n = 9) that were evaluated on days 1,4,9,13,17, 23 and 30. * P < 0.05, **P < 0.01 compared to the control group. # P < 0.05, ##P < 0.01, compare to the levodopa group, & P < 0.05 and &&P < 0.01 compared the results of AIMs on day 30 with the day 23 in each group.

Levodopa treatment started at high dose (50 mg/kg/day. i.p) to induce dyskinesia. AIMs test was performed to evaluate dyskinesia. The results showed that in the first group (levodopa group) on day 9, AIMs scores increased significantly compared to the control group (P < 0.01) indicating that dyskinesia has occurred (Fig. 2). AIMs scores' results for groups that recieved levodopa + ondansetron 0.04 and 0.08 mg/kg/day showed significant decrease compared to the levodopa group (P < 0.01). However, AIMs scores for these two groups were more than the control group almost in all days. Continuing treatment and evaluations on days 13, 17 and 23 more decrease in AIMs scores were observed in groups treated with ondansetron. More decrease was recorded in the group receiving higher dose of ondansetron (0.08 mg/kg/day), although was not significant. AIMs scores significantly increased in the first group that treated with levodopa till the end of the experiment (day 30). On day 23, ondansetron administrations in two groups were discontinued and the AIMs scores were measured on day 30. Interestingly, the scores increased in both groups, significantly compared to day 23 (P < 0.01).

### 3.3 Rotarod behavioral test results

**Fig. 3:**
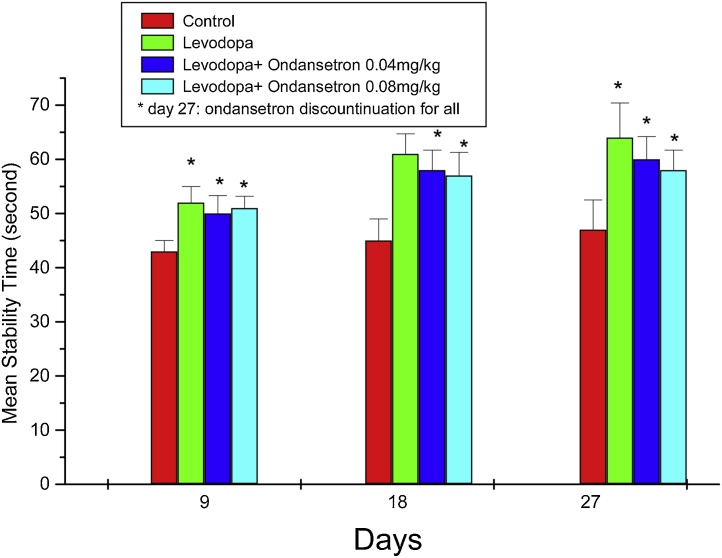
Mean stability scores ± SEM in rotarod for four groups (n = 9) that are evaluated on days 9, 18 and 27. * P < 0.05 compared to the control group.

Rotarod test results that are shown in Fig. 3, comparing the motor stability of rats in rotarod tool in four groups on days 9, 18 and 27 after treatments. Mean endurance of rats in rotarod tool that were treated with levodopa or levodopa + ondansetron increased by time when compared to the control group (P < 0.05). The highest mean scores were recorded in the levodopa group on day 27 after treatment. No significant differences can be reported among treated groups.

## 4 Discussion

In this study, we observed that early treatment with levodopa at 50 mg/kg could increase AIMs scores; the test that is normally use for dyskinetic measurements in animals (axial, limb, orolingual and locomotion scores). Continuing levodopa treatment (9 days), scores increased time-dependently. By adding ondansetron to levodopa in separate groups, all scores were reduced and stayed low till the last session of treatment (23 days).

Based on AIMs' results in groups with chronic administration of ondansetron + levodopa, that was even higher than the control group and lower than the levodopa treated group, it might be concluded that anti-dyskinetic effects has been observed. Upon discontinuations of ondansetron in two groups, AIMs scores increased significantly that confirm well its effectiveness in dyskinesia.

On the other hand, in rotarod test, that indicates motor performance, the results did not show any differences between levodopa and levodpa + ondansetron groups, therefore ondansetron could not probably interfere with levodopa's anti-parkinsonian efficacy. The scores for all days measured, were the same.

Dopamine and serotonin neurotransmitters both manage mental and physiological processes and an imbalance in those chemicals can lead to mental and physical disorders. However, the interactions of those two is very complex and have received considerable attention due to improve disorders related to central dopamine neuron dysfunctions such as Parkinson's disease, schizophrenia, or drug abuse with serotonergic drugs. Some biochemical and behavioral data indicate that serotonin (5-HT) affects dopaminergic terminal function in the striatum. It is also suggested that the endogenous 5-HT system affects striatal DA release in a state-dependent manner associated with the conditional involvement of various 5-HT receptors such as 5-HT(2 A), 5-HT(2C), 5-HT(3), and 5-HT(4) receptors (Navailles and De Deurwaerdère, 2011).

In animal models of Parkinson’s disease, the serotonin system has appeared to play an important role in the induction of LID. It is believed and to some extent approved by some animal experiments that serotonin neurons have the enzymatic machinery able to convert exogenous levodopa to dopamine, and interfere with its storage and release without a feed-back control mechanism, so that are not able to regulate synaptic dopamine levels. In partial dopamine depletion, some dopamine terminals still can control dopamine released from serotonin neurons. But with the progression of dopamine neuron degeneration this protective mechanism impairs, causing fluctuations in synaptic dopamine levels and palpitate stimulation of post-synaptic dopamine receptors. Removal of serotonin neurons by selective toxin, or some pharmacological antagonists have been reported to produce complete suppression of LID in animal models of Parkinson’s disease (Carta et al., 2007; Carta & Trounchi, 2014). Dampening of serotonin neuron release by 5-HT1 receptor agonists have been reported to not only reduces LID, but it has also been shown to prevent induction of post-synaptic alterations at striatal neurons (Munoz et al., 2008).

In many previous studies, some 5 H T receptor interactions like blockade of 5 H T2, 5 H T3 and 5 H T6 receptors and stimulation of 5 H T1A receptors had been reported to be effective in decreasing bradykinesia and akynesia. (Carta et al., 2007; Ohno et al., 2011a,2011b; Guridi et al., 2012; Bonsi et al., 2007; Carta et al., 2008; Iravani et al., 2006; Bishop et al., 2012; Vanover et al., 2008; Lindenbach et al., 2015; Carlsson et al., 2007; Politis et al., 2014; Ohno et al., 2011a,2011b; Ohno et al., 2013a,2013b).

Based on a review report; serotonin has a role in regulating dopamine release via 5-HT3 receptors (Dremencove et al., 2006) which is one of the reasons for dyskinetic phenomenon. Therefore, considering our results, we assume that blockade of serotonin stimulation via 5-HT3 antagonists (like ondansetron) suppress or modulate excessive and uncontrollable release of dopamine.

The therapeutic effects of ondansetron in psychotic states induced by levodopa and improving tardive dyskinesia caused by typical antipsychotic drugs are well established and implicated that serotonin is one of the main regulators of dopamine release in striatal region; therefore it can affect dyskinetic movements (Zoldan et al., 1995; Sirota et al., 2000; Stockmeier et al., 1993).

An increase in dopamine can stimulate serotonin release in brain, as well. Although neither block nor stimulation of the 5 H T3 receptor had effects on parkinsonian tremor (Costall et al., 1987, 1990; Kawashima et al., 2013) mainly because the degeneration largely occurs prior to the onset of symptoms.

In this study we did not use benserazide that is usually given to prevent peripheral side effects of levodopa. In fact first we thought that if animals could tolerate levodopa alone, there is no need to add it (and it was true). Second, because the major peripheral side effect of levodopa is nausea that is well prevented by ondansetron.

In conclusion, AIMs measurements are reported to be one of the most important key features of LID. It provides a basis for dyskinesia severity ratings in animal models (Lundblad et al., 2005). However the rotarod behavioral test often uses to measure movement performance in parkinson's disease. In this study, the impact of ondansetron on dyskinesia is well documented. While blockade of 5 H T3 receptor with ondansetron caused a decrease in AIMs scores, it had no effects on rats' motor performance in rotarod behavioral test. It can be interpreted that using both tests can discriminate between different movement disorders.

Because of widespread availability of ondansetron and its well-known therapeutic effect as a potent anti-nausea without any major side effects (Berger et al., 2009), it can be a good adjunct agent with levodopa to postpone LID.

In order to highlight the mechanisms underlying these findings, the study of the expression of serotonergic projections and its related receptors (5-HT3) in the striatum regions after LID induction, are valuable.

## Conflicts of interest

The author reports no conflicts of interest in this work.
